# Systematic Review of the Neurobiological Relevance of Chemokines to Psychiatric Disorders

**DOI:** 10.3389/fncel.2015.00357

**Published:** 2015-09-10

**Authors:** Michael J. Stuart, Gaurav Singhal, Bernhard T. Baune

**Affiliations:** ^1^Psychiatric Neuroscience Lab, Discipline of Psychiatry, University of Adelaide, Adelaide, SA, Australia; ^2^School of Medicine, University of Queensland, Brisbane, QLD, Australia

**Keywords:** depression, Alzheimer’s disease, neurogenesis, chemokine, schizophrenia, inflammation, immune, neurodegeneration

## Abstract

Psychiatric disorders are highly prevalent and disabling conditions of increasing public health relevance. Much recent research has focused on the role of cytokines in the pathophysiology of psychiatric disorders; however, the related family of immune proteins designated chemokines has been relatively neglected. Chemokines were originally identified as having chemotactic function on immune cells; however, recent evidence has begun to elucidate novel, brain-specific functions of these proteins of relevance to the mechanisms of psychiatric disorders. A systematic review of both human and animal literature in the PubMed and Google Scholar databases was undertaken. After application of all inclusion and exclusion criteria, 157 references were remained for the review. Some early mechanistic evidence does associate select chemokines with the neurobiological processes, including neurogenesis, modulation of the neuroinflammatory response, regulation of the hypothalamus–pituitary–adrenal axis, and modulation of neurotransmitter systems. This early evidence however does not clearly demonstrate any specificity for a certain psychiatric disorder, but is primarily relevant to mechanisms which are shared across disorders. Notable exceptions include CCL11 that has recently been shown to impair hippocampal function in aging – of distinct relevance to Alzheimer’s disease and depression in the elderly, and pre-natal exposure to CXCL8 that may disrupt early neurodevelopmental periods predisposing to schizophrenia. Pro-inflammatory chemokines, such as CCL2, CCL7, CCL8, CCL12, and CCL13, have been shown to drive chemotaxis of pro-inflammatory cells to the inflamed or injured CNS. Likewise, CX3CL has been implicated in promoting glial cells activation, pro-inflammatory cytokines secretion, expression of ICAM-1, and recruitment of CD4+ T-cells into the CNS during neuroinflammatory processes. With further translational research, chemokines may present novel diagnostic and/or therapeutic targets in psychiatric disorders.

## Introduction

Psychiatric disorders are highly prevalent and disabling conditions of increasing public health relevance (Whiteford et al., 2013). Despite the global significance of these disorders, many patients remain resistant to current psychosocial and pharmacological interventions (Murdoch and Finn, 2000; Ono et al., 2003; Rush et al., 2006a,b; Trivedi et al., 2006). Contemporary advances in neuroscience are yet to successfully translate into improved clinical outcomes for these patients; however, there remains optimism within the research community that a greater translational focus may provide the necessary platform for development of novel therapeutics (Licinio, 2011). One area of neuroscience which has yet to receive such a translational approach is the increasing recognition of central nervous system (CNS)-specific mechanisms of the immune proteins designated as chemokines. The bulk of literature within the field of psychoneuroimmunology has concentrated on the application of detrimental (or more recently, beneficial) effects of immune cells and soluble mediators to the CNS through their canonical immune functions. For example, the putative role of pro-inflammatory cytokines and cells in degenerative processes which may be relevant to depressive disorders is analogous to their functions in the systemic immune system (Dantzer et al., 2008; Eyre and Baune, 2012). Discovery of chemokines extends back to as early as 1977 (Walz et al., 1977; Wu et al., 1977; Callewaere et al., 2007), however their role in modifying the neuroimmune and neurobiological processes received attention not until mid-90s (Tani and Ransohoff, 1994).

Chemokines were initially described as chemotactic factors regulating the migration of peripheral immune cells – an action which is likely relevant to the aforementioned pro-inflammatory cascades (Murphy et al., 2000). In addition to chemotaxis, chemokines have been described to have potentiating and activating actions on peripheral immune cells directing them to a pro-inflammatory activation state which may contribute to the neurodegenerative and pro-apoptotic cascades described in depression and Alzheimer’s disease (AD) (Ono et al., 2003; Le et al., 2004; Moylan et al., 2012; Jo et al., 2014). For example, CCL11 has been described as a key blood-borne factor, which is responsible for the aging-associated impairment in both hippocampal neurogenesis, and functional learning and memory (Villeda et al., 2011). Early evidence has begun to emerge suggesting novel non-immune and CNS-specific mechanisms of chemokines, including neuromodulation, neuroendocrine regulation, and direct neurotransmitter-like actions (Rostene et al., 2007, 2011a; Reaux-Le Goazigo et al., 2013). Moreover, chemokine receptor knockout mice (CCR6, CCR7, CXCR5) have recently been described to exhibit behavioral and neurobiological phenotypes of relevance to psychiatric disorders, which therefore may be of value as animal models of certain psychiatric symptoms (Harrison et al., 2014; Jaehne and Baune, 2014; Stuart et al., 2014).

We have recently systematically reviewed clinical studies of the association between chemokines and psychiatric disorders, including depression, bipolar disorder, schizophrenia, mild cognitive impairment, and AD – finding that alteration in serum or cerebrospinal fluid levels of many chemokines are broadly associated with psychiatric disorders irrespective of diagnostic category (Stuart and Baune, 2014). Taken together, the relevance of the aforementioned actions of chemokines to neurobiological mechanisms previously implicated in many psychiatric disorders, coupled with clinical evidence demonstrating significant differences in the expression of these chemokines broadly across the spectrum of psychiatric disorders are suggestive of a potential pathologically pertinent role of these factors in these disorders. Although currently there is little clinical evidence of differential profiles of chemokine expression in different psychiatric disorders, the mechanisms by which chemokines contribute to the pathogenesis and/or pathophysiology of these disorders may be disparate. For example, the relevance of the chemokine CCL11 as discussed above to hippocampal neurobiology in aging may be more relevant to AD and the second peak of depression incidence in older age – both of which are associated with hippocampal pathology (Villeda et al., 2011; Baruch et al., 2014). Likewise, elevated levels of maternal CXCL8 has been implicated in increased risk of psychosis in offspring, likely due to disruption of early neurodevelopment, in keeping with the suggestion that schizophrenia may be a disorder of neurodevelopmental origin (Brown et al., 2004). At a mechanistic level, CXCL8 has both gross pro-inflammatory actions which may contribute to pathological cascades within the CNS as reviewed below but also has a chemotactic function in guiding the apoptosis and/or migration of neural progenitor cells – the deregulation of which may be of pathological significance in periods of neural development (Kelland et al., 2011). Indeed, fetal exposure to CXCL8 has been associated with structural brain abnormalities in adulthood (Ellman et al., 2010).

Although aforementioned examples have clear disease-specific relevance for most chemokines, there is little specific data which imply pathotropism for a specific clinical entity. In light of the paucity of data on most chemokines, this review will focus on shared mechanisms of significance across clinical categories, such as neuroendocrine dysregulation, pro-inflammatory state and neurodegeneration, neurogenesis, and neurotransmitter system dysregulation. This review aims to systematically evaluate the immune and non-immune mechanisms by which chemokines may contribute to the pathophysiology or pathogenesis of psychiatric disorders both in adulthood and early neurodevelopmental periods. This is the first review to draw together both mechanisms analyzing combined effects with the purpose of illuminating areas of opportunity for further translational research. Given the aim of this study to enhance translational research and the paucity of postmortem human data, the scope of the review includes both rat/murine data and studies of human tissue where possible.

## Materials and Methods

**Figure 1:**
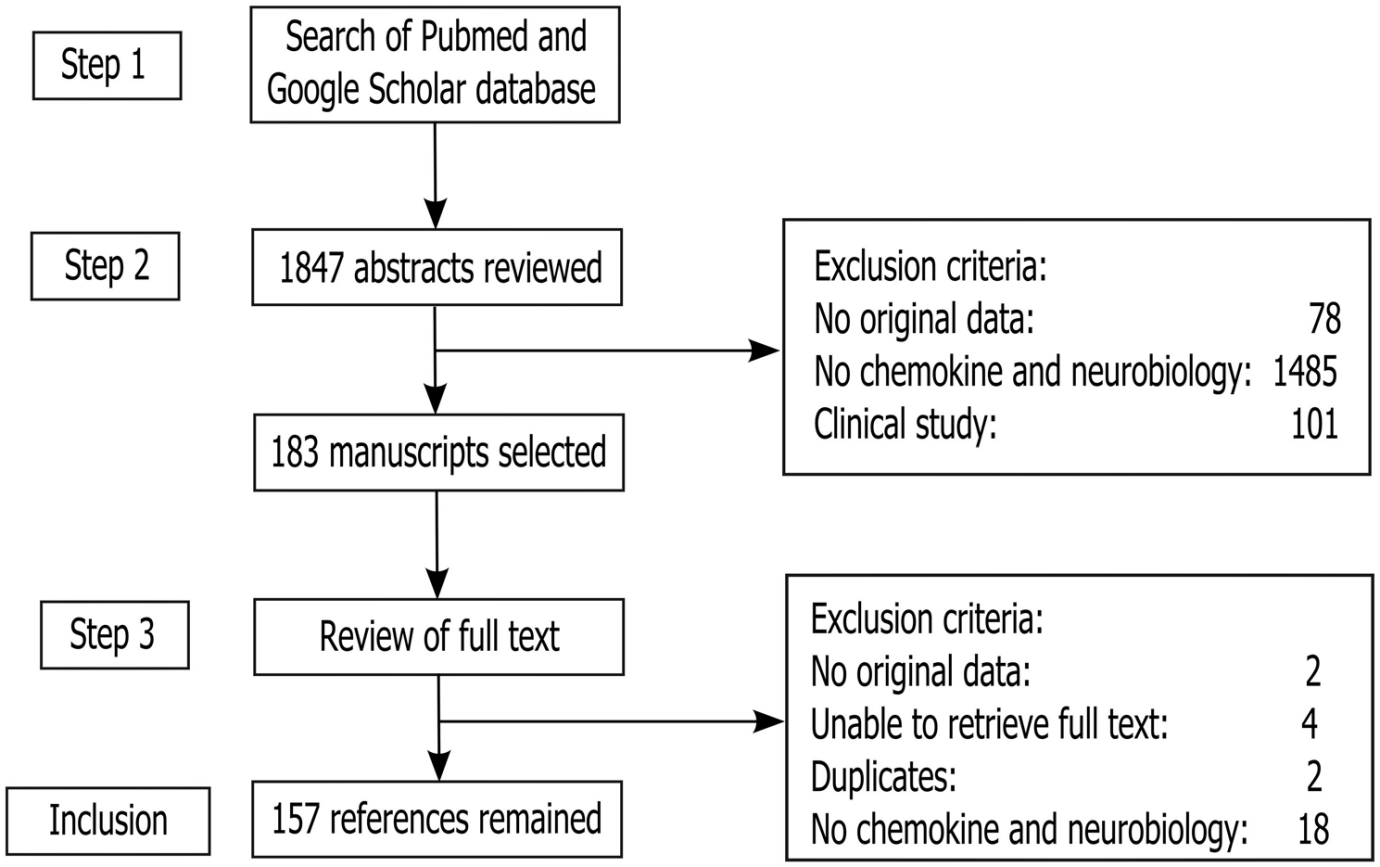
**Study inclusion flowchart**. It depicts the methodology for search and collection of relevant articles for this review, following PRISMA guidelines (Liberati et al., 2009; Moher et al., 2010).

The literature search for this review was carried out according to the PRISMA (Preferred Reporting Items for Systematic Reviews and Meta-analyses) guidelines as they apply to systematic reviews (Liberati et al., 2009; Moher et al., 2010). The checklist items from PRISMA as relevant to this review, for example those related to search and writing approaches, were included and the items not relevant, for example those related to meta-analyses, were excluded. The search strategy is included in Appendix. A total of 183 full text manuscripts were retrieved. Both human and rodent data were included. At this stage, 26 studies were excluded following the exclusion criteria as per Figure 1. In all, 157 articles remained for this review.

For clarity, all chemokines in this paper will be referred in agreement with the systematic nomenclature based on the position of their conserved cysteine residues as approved by the International Union of Pharmacology (Murphy et al., 2000). A list of common synonyms for all chemokines included in this review is provided in Table 1, along with a brief summary of their canonical functions in systemic immunity [see, for reviews, Murdoch and Finn (2000), Ono et al. (2003), and Le et al. (2004)].

**Table 1:** **Nomenclature and biological characteristics of chemokines [compiled from reviews (Murdoch and Finn, 2000; Ono et al., 2003; Le et al., 2004)]**.

Chemokine nomenclature	Receptor(s)	Synonyms	Classical peripheral functions^a^	CNS functions
**CC FAMILY**				
CCL1	CCR8	I-309, TCA-3	mø, B Lø, DC chemotaxis	Unknown
CCL2	CCR2	MCP-1	mø, T Lø, DC chemotaxis, and activation	NSC/NPC chemotaxis and differentiation Microglial phenotype modulation ?HPA axis modulation
CCL3	CCR1	MIP-1α	nø chemotaxis and activation	NSC/NPC, microglial chemotaxis
CCL4	CCR1 CCR5	MIP-1β	mø, T Lø, NK chemotaxis	Microglial chemotaxis
CCL5	CCR5	RANTES	T Lø, bø, eø chemotaxis, and activation	Microglial chemotaxis ?HPA axis modulation
CCL7	CCR2	MARC, MCP-3	mø chemotaxis	Unknown
CCL11	CCR2 CCR3 CCR5	Eotaxin	eø, bø chemotaxis	Impairs neurogenesis ?mechanism
CCL13	CCR2 CCR3 CCR5	MCP-4, NCC-1	mø, T Lø, bø, eø chemotaxis	Unknown
CCL15	CCR1 CCR3	Leukotactin-1, MIP-5, HCC-2, NCC-3	mø, T Lø, nø chemotaxis	Unknown
CCL18	GPR30	MIP-4, PARC	T Lø chemotaxis	Unknown
CCL20	CCR6	ELC, Exodus-3, Ckβ11	T Lø, nø, DC chemotaxis	T Lø chemotaxis
CCL24	CCR3	Eotaxin-2, MPIF-2, Ckβ6	T Lø, eø, bø, nø chemotaxis	Unknown
CCL25	CCR9	TECK	mø, T Lø, DC chemotaxis	Unknown
CCL26	CCR3	Eotaxin-3, MIP-4α, IMAC, TSC-1	eø, bø chemotaxis and activation	Unknown
CCL27	CCR10	CTACK, ILC, Eskine, Skinkine	T Lø chemotaxis	Unknown
**CXC FAMILY**				
CXCL1	CXCR2	Gro-α, GRO1, NAP-3, KC	nø chemotaxis	NSC/NPC chemotaxis and differentiation
CXCL2	CXCR2	Gro-β, GRO2, MIP-2α	nø chemotaxis	Unknown
CXCL8	CXCR1 CXCR2	IL-8, NAP-1, MDNCF, GCP-1	nø, eø, bø, T Lø, B Lø, NK, DC chemotaxis nø, mø, bø activation	NSC/NPC chemotaxis ?HPA axis modulation
CXCL9	CXCR3	MIG, CRG-10	T Lø chemotaxis	NSC/NPC differentiation PIC infiltration
CXCL10	CXCR3	IP-10, CRG-2	mø, T Lø, NK, DC chemotaxis	PIC infiltration
CXCL11	CXCR3 CXCR7	IP-9, I-TAC, β-R1	T Lø chemotaxis	PIC infiltration
CXCL12	CXCR4 CXCR7	SDF-1, PBSF	T Lø, mø chemotaxis Inhibit hematopoietic stem cell proliferation + differentiation Promote angiogenesis	NSC/NPC chemotaxis Enhance neurogenesis ?mechanism Modulate glutamate + GABA neurotransmission ?direct neurotransmitter-like effects ?HPA axis modulation
**CX3C FAMILY**				
CX3CL1	CX3CR1	Fractalkine, Neurotactin, ABCD-3	mø, T Lø, chemotaxis, and endothelial adhesion NK activation	Regulate microglial activation state

*^a^This list is not exhaustive but includes the classical functions associated with each chemokine*.

*mø, monocyte/macrophage; Lø, lymphocyte; nø, neutrophil; bø, basophil; eø, eosinophil; DC, dendritic cell; NK, natural killer cell; NSC/NPC, neural stem/progenitor cell; PIC, peripheral immune cell; HPA axis, hypothalamus–pituitary–adrenal axis*.

*? represents Unknown*.

## Chemokines in the CNS

### Introduction to chemokines

The term “chemokine” is a portmanteau of “chemotactic cytokine” and first coined in 1992 to accommodate a growing list of related proteins with chemotactic functions (Murphy et al., 2000). With the notable exception of CX3CL1 which has a membrane bound form, these proteins are present in a secreted soluble form. Receptors for these chemokines are primarily located on leukocyte subsets, and many receptors bind multiple ligands with variable affinity (Cyster, 1999; Murphy et al., 2000). Chemokines act through common intracellular signaling mechanisms to increase intracellular calcium (Nelson and Gruol, 2004) and their direct signaling is through G-protein-coupled receptors (Baggiolini et al., 1997). These chemotactic proteins are known to be important for leukocyte migration and activation under both physiological and pathological conditions. These processes are vital to physiological immune surveillance as well as inflammatory responses. Chemokines have also long been recognized to have additional functions, including inducing the release of pro-inflammatory mediators and control of T-helper (T_h_)-1/T_h_-2 phenotypic polarization (Cyster, 1999). Indeed, they have been categorized functionally into homeostatic and inflammatory chemokines. The former being always expressed in constitutive levels in certain organs and tissues and are required for basal immune cells migration, for example migration of the dendritic cells to the local-draining lymph nodes, where they activate more antigen-specific T cells. The latter, on the other hand, are expressed under the influence of pro-inflammatory factor, for example LPS, TNF-α, IL-1β) and further participate to upregulate the inflammatory response by attracting immune cells (e.g., macrophages, fibroblasts, T cells) to the site of inflammation (Rossi and Zlotnik, 2000; Zlotnik and Yoshie, 2000).

In this review, we will first describe the cellular and regional expression of chemokines and receptors before highlighting their roles in neurobiological processes implicated in the pathogenesis and pathophysiology of psychiatric disorders.

### Chemokines receptor and ligand expression in CNS by cell type

The chemokine receptors and their ligands are broadly expressed throughout both the developing and adult CNS [see, for reviews, Bajetto et al. (2001), Miller et al. (2008), Rostene et al. (2011b), and Jaerve and Muller (2012)]. Several of these chemokines are constitutively expressed under normal conditions, including CCL2, CCL3, CCL19, CCL21, CXCL8, CXCL12, and CX3CL1 (Jaerve and Muller, 2012). Other chemokines are upregulated in response to injury or inflammation. Chemokines and receptors expressed in the CNS under either basal or inflammatory conditions are listed by cell type in Table 2. It can be appreciated from this table that the capacity to express chemokine receptors is the rule rather than the exception for most CNS cell types.

**Table 2:** **Chemokine receptor expression in the CNS (both human and rodent)**.

Chemokine receptor	Microglia	Astrocyte	Oligodendrocyte	Neuron	NSC/NPC^a^	Reference
CC FAMILY						
CCR1	+	+	+	±	+	Meucci et al. (1998), Nguyen et al. (2003), Eltayeb et al. (2007), Tran et al. (2007), Kan et al. (2012)
CCR2	+	+	−	+	+	Banisadr et al., 2002a, 2005, Tran et al. (2007)
CCR3	+	+	+	+	+	van der Meer et al. (2000), Flynn et al. (2003), Krathwohl and Kaiser, 2004
CCR4	+	+		+		Meucci et al. (1998), Flynn et al. (2003)
CCR5	+	+	−	−	+	Spleiss et al. (1998), Nguyen et al. (2003), Ji et al. (2004), Eltayeb et al. (2007), Tran et al. (2007), Kan et al. (2012)
CCR6	+	+		−		Coughlan et al. (2000), Flynn et al. (2003)
CCR7	+	+		+		Dijkstra et al. (2006), Liu et al. (2007), Gomez-Nicola et al. (2010)
CCR8	+	+		+		Trebst et al. (2003), Liu et al. (2007)
CCR9	+	+		+		Liu et al. (2007), de Haas et al. (2008)
CCR10	−	+		+		Flynn et al. (2003), Liu et al. (2007)
CXC FAMILY						
CXCR1	+	+	+	+	+	Puma et al. (2001), Flynn et al. (2003), Omari et al. (2005), Weiss et al. (2010)
CXCR2	+	+	+	+	−	Giovannelli et al. (1998), Flynn et al. (2003), Omari et al. (2005), Weiss et al. (2010)
CXCR3	+	+	+	+	+	Coughlan et al. (2000), Flynn et al. (2003), Omari et al. (2005), Tran et al. (2007)
CXCR4	+	+	+	+	+	Banisadr et al. (2002b, 2011a), Tran et al. (2007), Gottle et al. (2010)
CXCR5	+	+		+	+	Petito et al. (2001), Flynn et al. (2003), Bagaeva et al. (2006), Weiss et al. (2010)
CXCR7	−	+	+	+	±	Schonemeier et al. (2008), Gottle et al. (2010)
CXCL14 receptor (unidentified)					+	Banisadr et al. (2011b)
CX3C FAMILY						
CXCR1	+	+		+	+	Meucci et al. (1998), Ji et al. (2004), Sunnemark et al. (2005)

*^a^Neural stem/progenitor cells (NSC/NPC) isolated from either hippocampal or subventricular zone populations*.

The regulation of the expression of chemokines and their receptors involves a complex and poorly understood interplay between several systems. For example, in paradigms of CNS injury or inflammation, such as experimental autoimmune encephalomyelitis (EAE), the expression of chemokines and chemokine receptors may be increased through several mechanisms (Ubogu et al., 2006). These mechanisms include increase in their expression on lymphocytes from the cerebrospinal fluid and T cells that migrate across the blood-brain barrier (BBB), as well as on glial cells within the brain, particularly astrocytes. Likewise, while CX3CR1-deficiency in microglia has been shown to enhance beneficial microglial activity, increase amyloid clearance, and prevent neuron loss in mice models of AD (Harrison et al., 1998; Fuhrmann et al., 2010; Liu et al., 2010), this has also been shown to dysregulate microglial response and increase neurotoxicity following peripheral lipopolysaccharide injections in the CX3CR1 KO mice (Cardona et al., 2006), and decrease neurotoxicity with no harmful effects on microglia in mice models with focal cerebral ischemia (Dénes et al., 2008) or have no neurotoxic effects at all in neuroinflammatory conditions other than AD in mice (Jung et al., 2000). By contrast, CCR2 deficiency in microglia aggravated amyloid deposition; possibly due to the decreased migration and recruitment of inflammatory monocytes to the site of amyloid deposition in a transgenic mouse model of AD (Naert and Rivest, 2011). These examples are clearly suggestive of the complex mechanisms associated with chemokines and their receptors in the CNS.

Classical immune mediators are known to regulate the expression of chemokines both peripherally and in the CNS. For example, the T_h_1 cell-derived cytokine interferon-γ is known to induce the expression of the chemokines CXCL9, CXCL10, and CXCL11: the ligands for CXCR3 (Shurin et al., 2007). Similarly, a study showed that CCR8 failed to express with no rapid onset of inflammation in TNF KO mice models of EAE, suggesting TNF derived from the infiltrating hematopoietic cells is essential for the expression of CCR8 by the resident microglia (Murphy et al., 2002). Moreover, IL-33 released from damaged oligodendrocytes during CNS injury has recently been shown to induce expression of monocyte attracting chemokines, such as CCL2 and CXCL10, on local astrocytes and microglia (Gadani et al., 2015). Notably, IL-33, a member of IL-1 family of cytokines, is produced by intracellular complexes called as inflammasomes, commonly seen in glial cells, including microglia and astrocytes (Singhal et al., 2014). Other studies have also shown expression of IL-33 by glial cell, primarily astrocytes (Foster et al., 2015; Pomeshchik et al., 2015), which therefore suggests that expression of cytokines and chemokines on glial cells may be inter-regulated. Several neurotrophins also regulate the expression of certain chemokine receptors. It has been shown that brain-derived neurotrophic factor (BDNF), nerve growth factor (NGF), and neurotrophin-3 (NT-3) can regulate the expression of the chemokine receptors CXCR3, CXCR4, and CCR5 in the brain (Ahmed et al., 2008; Avdoshina et al., 2011). These receptors were selected for study due to their relevance to human immunodeficiency virus (HIV)-related dementia; however, further investigation of the capacity for neurotrophins to regulate chemokine receptors is warranted, given the reciprocal functions of chemokines in regulating neurotrophic processes.

## The Mechanistic Relevance of Chemokines to the Pathophysiology and Pathogenesis of Psychiatric Disorders

### Regulation of neurogenesis by chemokines

In recent years, a major focus of biological psychiatry research has been the processes of neurogenesis by which new neurons are generated, differentiated, and integrated into functional circuits. This is relevant to psychiatric disorders both in the context of early neurodevelopmental periods, and in adult neurogenesis – particularly in the hippocampal dentate gyrus. The disruption of early neurodevelopment has been implicated in many psychiatric disorders, particularly schizophrenia where a number of pre-natal maternal environmental factors have been proposed as risk factors for development of this disorder – for example smoking, infections, maternal mental illness, and stressful life events (Hunter et al., 2011; Betts et al., 2014a,b). An increasing body of both animal and human data has also implicated dysregulation of adult hippocampal neurogenesis in the pathophysiology of several psychiatric disorders, including depressive disorders and anxiety disorders (Eisch and Petrik, 2012; Eyre and Baune, 2012), while enhancement of hippocampal neurogenesis has been associated with several clinically efficacious treatment modalities, including exercise, omega-3 fatty acids, electroconvulsive therapy, and conventional antidepressant pharmacotherapy (Eisch and Petrik, 2012; Kang and Gleason, 2013; Moylan et al., 2013a; Dukart et al., 2014; Smitha et al., 2014). Some early evidence has begun to implicate several chemokines in processes relevant to neurogenesis both in early neurodevelopmental periods and adult neurogenic niche as discussed below.

#### CC Chemokines

Similar to their roles as chemotactic factors for immune cells, chemokines are involved in the regulation of neural stem/progenitor cell (NSC/NPC) migration in both endogenous neurogenic niches of the adult brain [hippocampus and subventricular zone (SVZ)] and exogenous (transplanted) cells to sites of lesions. We will not consider the regulation of exogenous stem cell migration here, as this is discussed in several recent reviews [see, for reviews, Martino et al. (2011), Jaerve and Muller (2012), and Kokaia et al. (2012)]. Many chemokines are known to have receptors expressed on NPC/NSCs derived from the SVZ or hippocampus (Table 2), and several of these have been shown to exert chemotactic effects on these cells *in vitro* through modified Boyden chamber assays. These include CCL2, CCL3, CXCL1, CXCL8, and CXCL12 (Imitola et al., 2004; Widera et al., 2004; Gordon et al., 2009; Kelland et al., 2011). In addition, several of these chemokines are implicated in *in vivo* migration of these cells to the site of chemically induced experimental injury (Gordon et al., 2009).

#### CXC Chemokines

From available evidence, it appears that most chemokine or chemokine receptor knockout mice are viable and demonstrate no obvious neural deficit, likely owing to the aforementioned significant redundancy in receptor–ligand interactions across chemokines (Bajetto et al., 2001). A notable exception is CXCL12 and its receptor CXCR4, where knockouts of either in mice result in a grossly malformed cerebellum with the absence of foliation secondary to aberrant premature migration of granular cells and a non-viable phenotype (Ma et al., 1998). Moreover, we have also recently demonstrated for the first time an enhancement in adult hippocampal neurogenesis with a relevant behavioral phenotype in a chemokine receptor knockout mouse, CXCR5^−/−^ (ligand CXCL13) (Stuart et al., 2014). However, in the latter case, the specific mechanisms underlying this phenotype remain unclear. Although no major deficits have been described in other chemokine/receptor knockouts, chemokines have been described to influence relevant underlying processes, such as neuronal/glial migration, proliferation, and differentiation (Zou et al., 1998; Stumm and Höllt, 2007; Turbic et al., 2011).

#### CX3C Chemokine: CX3CL1

As sated above, chemokines may have additional actions on NPC/NSCs, including the regulation of proliferation and differentiation. Of the chemokines involved in supporting neurogenesis, CX3CL1 is one of the most studied. This chemokine is highly expressed on mature neurons and astrocytes, and its receptor CX3CR1 is mostly expressed on microglia, with expression on mature neurons noted as well (Hatori et al., 2002; Ji et al., 2004; Kim et al., 2011; Vukovic et al., 2012). This chemokine has been shown to have multiple actions in the CNS of rodents, including regulation of microglial activation state (Cardona et al., 2006), microglial synaptic pruning of mature neurons (Paolicelli et al., 2011), and modulation of several neurotransmitter systems (Ragozzino et al., 2006; Heinisch and Kirby, 2009; Piccinin et al., 2010). Interestingly, CX3CL1 has been shown to decrease with aging in the male Fisher rats and is associated with the age-related suppression of neurogenesis (Bachstetter et al., 2011). Direct exogenous replacements of CX3CL1 or exercise-induced CX3CL1 have been shown to act via microglia to enhance *in vivo* neurogenesis and *in vitro* NSC/NPC activity (Bachstetter et al., 2011; Vukovic et al., 2012).

#### A Short Note on the Role of Above Three Families of Chemokines in Inducing Neurogenesis

This indirect enhancement of neurogenesis by CX3C chemokines via modulation of microglial phenotype (as mentioned above) may be relevant to the actions of other families of chemokines too. Indeed, other chemokines, such as CXCL12, have been shown to enhance neurogenesis, however the mechanism of this remains unclear (Wu et al., 2009). Conversely, the chemokine CCL11 has been implicated as mediator under the regulation of interferon which impairs hippocampal neurogenesis in the context of aging (Baruch et al., 2014). It is important to note conflicting findings regarding the *in vitro* effects of CX3CL1 and CXCL12, and CCL5, which have also been reported to impair proliferation in NPC/NSC cultures (Krathwohl and Kaiser, 2004). One potential mechanism of these effects may be that they are mediated through the regulation of neurotrophic factors (e.g., BDNF/NGF); however, to our knowledge no chemokines have been shown to influence the expression of neurotrophic factors in the CNS.

In addition to regulation of proliferation, several chemokines have a role in the regulation of NSC/NPC differentiation. Indeed, it has been shown that CCL2, CCL21, and CXCL9 favor neuronal differentiation, while CXCL1 and CXCL9 also favor oligodendrocyte differentiation in the adult mouse brain (Turbic et al., 2011). However, no chemokines have been noted to influence microglial or astroglial differentiation as of now.

### Regulation of CNS inflammatory state by chemokines: Neurodegenerative and neuroprotective effects

The most recognized functions of chemokines in the CNS are to regulate the inflammatory state associated with various pathological conditions, including paradigms of CNS injury such as ischemic stroke and trauma or autoimmune responses as in multiple sclerosis or EAE (Stefini et al., 2008). The functional contribution of chemokines in these states has been extensively reviewed elsewhere (Jaerve and Muller, 2012).

#### CC Chemokines

The monocyte chemotactic protein (MCP) family including CCL2, CCL7, CCL8, CCL12, and CCL13 (designated as MCP 1–5, respectively) exert potent pro-inflammatory actions through chemotaxis of monocyte-derived macrophages and other inflammatory leukocytes to the inflamed or injured CNS as seen after focal transient ischemia in rats (Yamagami et al., 1999). Of these chemokines, CCL2 is the most studied and has been shown to be selectively translocated across endothelial cells of the BBB enabling chemotaxis of circulating leukocytes (Weiss et al., 1998; Ge et al., 2008). CCL2 has also been shown to have similar effects on microglia, inducing migration and proliferation but did not directly induce a pro-inflammatory or neurotoxic phenotype (Hinojosa et al., 2011). Interestingly, CCL2 treatment of microglia may indirectly increase pro-inflammatory activity by increasing the migration of P2 × 4 purinergic receptors to the cell surface (Toyomitsu et al., 2012). Activation of these receptors by adenosine triphosphate (ATP) has been shown to induce the expression of pro-inflammatory cytokines by microglia (Inoue, 2006).

The chemokines CCL3, CCL4, and CCL5 have been shown to exhibit diverse chemotactic functions in the inflamed CNS, including actions on monocytes, microglia, and neutrophils via their receptors CCR1, CCR3, and CCR5 (Murphy et al., 2000; Cowell et al., 2002; Johnson et al., 2011). However, the effects of these chemokines in inflammatory states remain unclear, with divergent effects reported in different brain regions and models (Hau et al., 2008; Passos et al., 2009; Buschmann et al., 2012). For example, during early and late cuprizone-induced demyelination, the resultant microgliosis/astrocytosis appeared to be greater in the subcortical white matter tract corpus callosum than in the gray matter cortex region, in concurrent with the expression of the key chemokines CCL2 and CCL3 (Buschmann et al., 2012). Likewise, mononuclear cells derived from the human umbilical cord have been shown to cause increase of CCL3 and CCL5 chemokines in neurons with noticeable reduction in apoptosis (Hau et al., 2008). By contrast, MIP-1α and CCR5 have been shown to be essential for the accumulation of activated glial cells in the hippocampus of mice models of AD leading to inflammation and cognitive failure (Passos et al., 2009).

Although chemokines have potent functions in regulation of neuroinflammation in models of trauma or EAE, for most chemokines it remains to be studied whether their functions are also relevant to regulation of the chronic, low grade inflammatory state hypothesized to be relevant to psychiatric disorders. An exception to this is in the context of aging where CCL11 and CCL17 (as well as CXCL10) were demonstrated to be downstream mediators of the effects of systemic interferons on inflammatory state at the choroid plexus and correlated hippocampal neurogenesis and hippocampal-dependent learning and memory tasks (Villeda et al., 2011; Baruch et al., 2014). Aged mice demonstrate higher levels of CCL2, CCL11, CCL12, and CCL19 in association with deficits in hippocampus-dependent learning and memory tasks, *ex vivo* hippocampal slice electrophysiological induction of long-term potentiation, reduction in size and number of neurospheres (readout of NSC/NPC populations), and number of doublecortin positive, NeuN positive, and bromodeoxyuridine uptake positive immature neuronal cells (Villeda et al., 2011). The effects of aging on these parameters could be replicated by intracerebroventricular injection of recombinant CCL11, and the effects of aging could be prevented by systemic or intracerebroventricular administration of neutralizing antibodies for CCL11 (Villeda et al., 2011). Interestingly, however, parabiosis of a young animal to an aged animal was not sufficient to reverse the effects of aging on CCL11 expression at the choroid plexus, suggesting that under conditions of normal aging both systemic and brain-derived factors (including interferon-γ) may drive CCL11 expression (Baruch et al., 2014). Taken together, these findings implicate CCL11 as a key player in the systemic immune influence on hippocampal function – with great relevance to psychiatric disorders, particularly depressive disorders and AD.

#### CXC Chemokines

A major role of chemokines in regulation of CNS inflammatory states is the regulation of neutrophil chemotaxis. This is a major function of the chemokines CXCL1–CXCL8: ligands for CXCR2 which is highly expressed on neutrophils (Murphy et al., 2000). The overall beneficial/detrimental effect of these chemokines in enhancing or impairing neuronal survival and repair under inflammatory conditions remains unclear (Jaerve and Muller, 2012), as is the role of neutrophils themselves (Stirling et al., 2009). For example, CXCR2 and its ligand CXCL1 have been shown to exert neuroprotective effects in the mouse model of EAE (Omari et al., 2009), however the opposite effects have also been reported in a similar model (Kerstetter et al., 2009). Similarly, the chemokines CXCL1, CXCL2, and CXCL8 were shown to have neuroprotective effects in a model of β-amyloid toxicity *in vitro* (Watson and Fan, 2005), however *in vivo* neutralization of CXCL8 has been shown to mitigate neurological and histological deficits in a model of ischemic stroke (Villa et al., 2007). This suggests that complex differences related to both the specific disease pathology, and nuances of the temporal activities of different cell types targeted by these chemokines may each be relevant to their overall beneficial/detrimental effect [see, for reviews, Ubogu et al. (2006) and Mirabelli-Badenier et al. (2011)]. This would render these chemokines difficult therapeutic targets.

The chemokines CXCL9–CXCL11 have clearer detrimental pro-inflammatory effects through CXCR3-mediated chemotaxis of natural killer cells, T_h_1 cells, and their associated classically activated (M1) pro-inflammatory monocyte-derived macrophages (Murphy et al., 2000). For example, CXCL10 is known to be translocated across vascular endothelial cells from the CNS (Mordelet et al., 2007). Blockade of CXCR3 reduced the infiltration of the aforementioned cell types, reduced tissue damage, and reduced functional deficit in the mouse and rat models of EAE (Jenh et al., 2012), but selective knockout of CXCL10 did not impair the severity of EAE in mice – likely due to redundancy in functions with CXCL9 and CXCL11 (Klein et al., 2004).

#### CX3C Chemokine: CX3CL1

While CX3CL1 has been shown to be neuroprotective and upregulate in the CA1, CA3 and dentate gyrus of the rat hippocampus during synaptic scaling in the healthy brain of adolescent male Wistar rats (Sheridan et al., 2014), recent studies have provided an evidence for its role in promoting microglial and astrocytic activation, pro-inflammatory cytokines secretion, expression of intracellular adhesion molecule (ICAM-1) and recruitment of CD4+ T-cells into the CNS during neuroinflammatory processes, in particular multiple sclerosis and AD (Sheridan and Murphy, 2013; Blauth et al., 2015; Plese et al., 2015). In an *in vitro* study on frozen postmortem brain tissues from cases with different neuropathological states of AD and age-matched controls, an enhanced expression of CX3CL1 in brain regions with more vulnerability to AD-related changes, such as hippocampus, has also been noted where the level of CX3CL1 expression reflected the course of disease (Strobel et al., 2015). Moreover, CX3CL1 induced at the choroid plexus by the extracellular adenosine has been shown to trigger migration of lymphocytes into the CNS in mice models of EAE (Mills et al., 2012). Interestingly, randomized controlled trials on rodent models of AD have indicated that it is the membrane bound CX3CL1 and not its soluble form that regulate microglial phagocytosis of Aβ, as well as neuronal microtubule-associated protein tau (MAPT) phosphorylation (Lee et al., 2014), which when accumulated may result in instability of microtubules, consequent loss of effective transport of molecules and organelles, and ultimately neuronal death (KoSIK et al., 1986). However, a positive correlation between the plasma levels of soluble CX3CL1, and the course of AD and mild cognitive impairment has also been reported, providing an evidence for the involvement of soluble CX3CL1 in the pathogenesis of AD (Kim et al., 2008). For neuroinflammatory vs. neuroprotective effects of CX3CL1, please see review by Ferretti et al. (2014).

### Non-immune neuromodulatory activities of chemokines

Recent evidence has also begun to elucidate neurotransmitter-like and/or neuromodulatory actions of select chemokines, distinct from their immune function. This has been the subject of several recent reviews, however we will summarize the mechanisms of these neuron–neuron interactions for the most studied chemokine receptor examples [see, for reviews, Rostene et al. (2007, 2011b) and Melik-Parsadaniantz and Rostene (2008)]. These actions are best studied in the chemokine CXCL12 and its receptors CXCR4 and CXCR7; however, early evidence suggests similar roles for other chemokines, including CCL2, CCL5, and CCL21.

Chemokines have the capacity to modulate the release of classical neurotransmitters from neurons. For example, CCL5 has been shown to modulate the release of glutamate from both cortex and spinal cord in mice through interactions with its receptors CCR1 and CCR5 (Di Prisco et al., 2012). Likewise, CXCL12 has been shown to pre-synaptically regulate glutamatergic and GABAergic neurotransmission in the rat substantia nigra – resulting in modulation of their downstream dopaminergic neurons (Guyon et al., 2006). Another study on the male Wistar rats showed that CXCL12 may also have direct neurotransmitter-like post-synaptic effects on these dopaminergic neurons through interactions with CXCR4 expressed on those neurons (Skrzydelski et al., 2007). These findings may be relevant as therapeutic targets for the management of psychosis or the mitigation of extrapyramidal side effects of convention anti-psychotic pharmacotherapy. Similar effects of CXCL12 and CX3CL1 on glutamatergic and GABAergic signaling regulation of serotonergic neurons in the dorsal raphe nucleus of rats have also been demonstrated with evident potential relevance to mood disorders (Heinisch and Kirby, 2009, 2010).

Although direct signaling of chemokines is through G protein-coupled receptors, these may be linked to ionotropic receptors and other intracellular signaling pathways that converge to increase intracellular calcium – indicating a potential role in contributing to synaptic plasticity or indeed to excitotoxicity, however these functional roles remain speculative (Rostene et al., 2007, 2011b).

#### Regulation of Neuroendocrine Function by Chemokines

Early evidence for a role of chemokines in regulation of neuroendocrine function has also recently been reviewed (Rostene et al., 2011a; Verburg-van Kemenade et al., 2013). It has been suggested that several chemokines, most notably CCL2, CCL5, CXCL8, and CXCL12, are involved in the regulation of the hypothalamus–pituitary–adrenal (HPA) axis influencing each of the major neuroendocrine hormones and their broad physiological functions in stress, metabolisms, feeding behaviors, reproduction, and fluid/electrolyte balance. Considering the relevance of the HPA axis to psychiatric disorders and extensive use of rodent models in psychiatric research, we restrict our discussion to the HPA axis system in rodents.

Upregulation of pro-inflammatory chemokines, including CCL2 and CXCL1 both peripherally and in the CNS, occurs in response to various stressors, including peripheral LPS injection, intermittent cold stress, and immobilization stress (Girotti et al., 2011). On the contrary, the expression of these pro-inflammatory chemokines is down-regulated by glucocorticoids (Sorrells and Sapolsky, 2007; Zhou et al., 2007). There is some suggestion that chemokines may participate in the regulation of the HPA axis; however, this has not been studied *in vivo*. For example, CXCL8 is known to be expressed in the paraventricular nucleus – one region where negative feedback on the HPA axis is exerted (Licinio et al., 1992), and CXCL1 is known to stimulate release of adrenocorticotrophic hormone from cultured pituitary neurons (Sawada et al., 1994). It is therefore tempting to speculate that the pro-inflammatory chemokines may participate in the modulation of glucocorticoid receptor-mediated negative feedback in psychiatric disorders in a similar manner to other cytokines.

## Discussion

**Figure 2:**
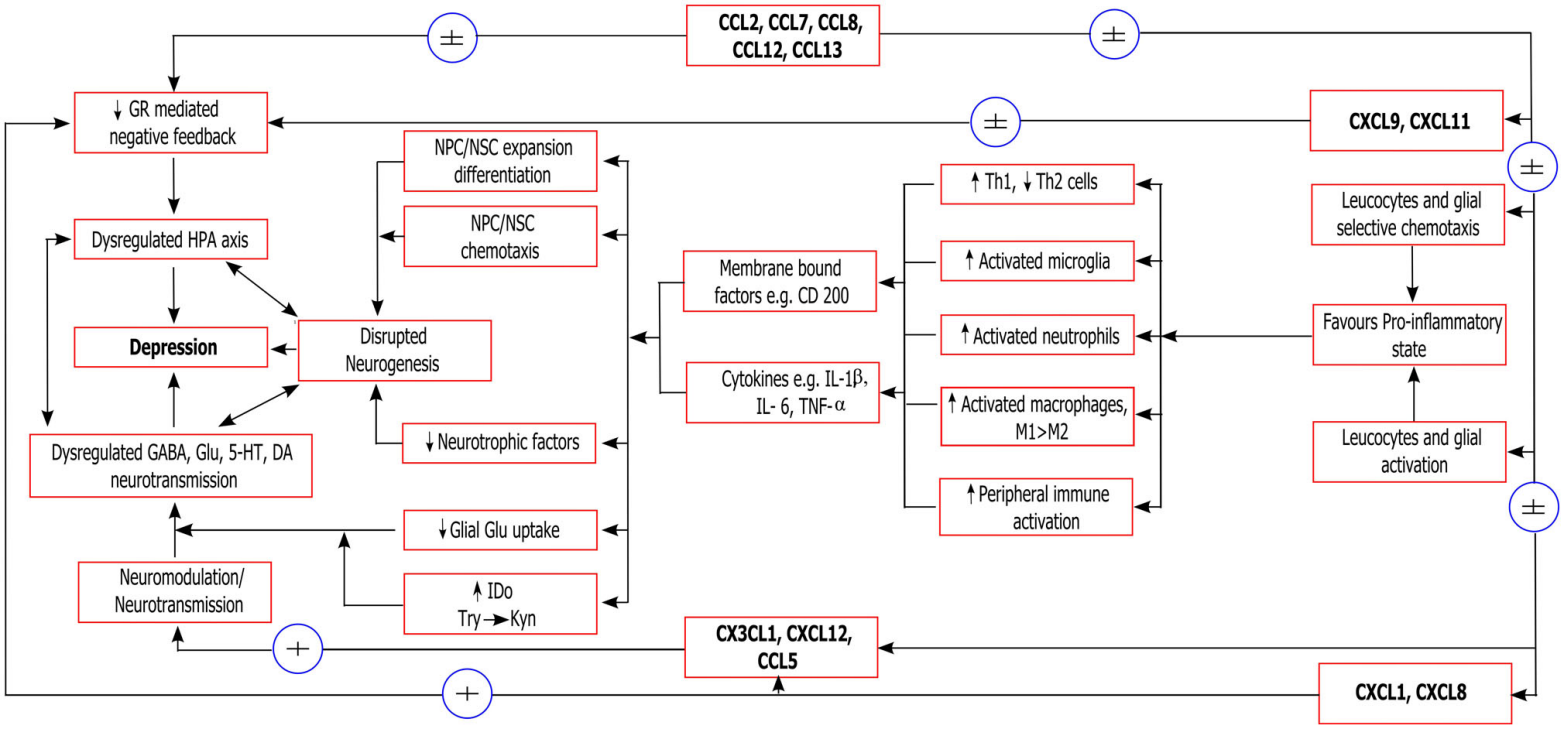
**Neurobiological mechanisms of chemokines relevant to depression**. ±, possible effect (weak/equivocal evidence of effect); + evidence of effect; GR, glucocorticoid receptor; HPA axis, hypothalamus/pituitary/adrenal axis; NPC/NSC, neural stem/progenitor cells; Th1/Th2, T-helper type 1/2 cells; M1/M2, classically activated/alternatively activated macrophages, respectively; BDNF, brain-derived neurotrophic factor; TrkB, receptor for BDNF; IDO, 2,3-indoleamine dioxygenase; Trp, tryptophan; KYN, kynurenine; Glu, glutamate; GABA, gamma aminobutyric acid; 5-HT, serotonin; DA, dopamine.

Given the emerging clinical evidence demonstrating an association between altered serum, CSF, and brain tissue levels of many chemokines and several major psychiatric disorders, it has become relevant to consider the possible mechanisms by which these factors may interact with known neurobiological processes which have been strongly implicated in the pathogenesis and pathophysiology of these disorders (Stuart and Baune, 2014). As we have described above, some early mechanistic evidence does associate select chemokines with the neurobiological processes, including neurogenesis, modulation of the neuroinflammatory response, regulation of the HPA axis, and modulation of neurotransmitter systems. As with the current clinical evidence, this early evidence does not clearly demonstrate any specificity for a certain psychiatric disorder, but is primarily relevant to mechanisms which are shared across disorders. In Figure 2, we present a prototypical model of the role of chemokines in a psychiatric disorder using the example of depression where chemokines interact with pathophysiological cascades implicated in the neurobiology of depressive disorders (Eyre and Baune, 2012; Stuart and Baune, 2012). These inter-linked cascades culminate in enhancement of the neuroinflammatory response, oxidative stress and apoptotic pathways, impairment of neurogenesis, loss of HPA axis regulation, and disruption of serotonergic and glutamatergic neurotransmission. In this figure, chemokines (e.g., CX3CL1, CXCL12, CCL5) with direct neurotransmitter-like or neuromodulatory actions may directly influence neurotransmitter systems that are implicated in depression. These neurotransmitter systems interact bidirectionally with neurogenesis and HPA axis dysregulation. Classical pro-inflammatory chemokines, including the CXCL1–8 family, directly activate leukocytes and glia to a pro-inflammatory state (Th1 T cells, M1 macrophages, activated glia) (Murphy et al., 2000), while indirectly contributing to loss of GR-mediated negative feedback on the inflammatory response (when chronic). Pro-inflammatory chemokines, including those with a primarily chemotactic activity (e.g., CCL2, CCL7, CCL8, CCL12, CCL13, CXCL9–11), may also contribute via selective chemotaxis of pro-inflammatory type cells (as above) to the CNS or choroid plexus (Yamagami et al., 1999). This selective recruitment of pro-inflammatory cells may drive the inflammatory response. The gross effects of a chronic pro-inflammatory state in contributing to clinical episodes of depression have been described elsewhere, however in brief will include an inter-linked impairment of neurogenesis, enhancement of neurotoxicity, disruption of neurotransmitter systems, dysregulation of the HPA axis, suppression of neurotrophic factors, and aberrant tryptophan metabolism (Dantzer et al., 2008; Moylan et al., 2013b).

It is relevant to note that all of these findings remain early, few chemokines have been investigated in each context, and each finding awaits independent replication. Of importance is the extensive expression of many chemokines and receptors on many cell types throughout the CNS which is suggestive of functional relevance that remains unappreciated (Table 2). Further study in all areas will therefore be required in order to draw any reliable conclusions regarding the neurobiological significance of the observed clinical associations between chemokines and psychiatric disorders. From the clinical literature, the most robust chemokine associations are noted for CXCL8 and CCL2 (Stuart and Baune, 2014), however outside of their classical inflammatory activities no reports are available regarding the presence or absence of CNS-specific biological functions of these chemokines. Although this absence of data may simply reflect the incomplete reporting of negative animal studies, it is relevant to consider these chemokines as promising candidates for future mechanistic investigations.

## Concluding Remarks

Previously chemokines have been relatively neglected family of immune proteins in investigations of CNS-immune axis dysfunction in psychiatric disorders. Early clinical evidence has however begun to associate chemokines with psychiatric disorders, irrespective of current diagnostic category. Similarly, early mechanistic evidence provides plausible mechanisms by which these chemokines may contribute to the pathophysiology of these disorders, however both these clinical and basic science results will require further replication. With further study, chemokines may become relevant as novel diagnostic markers or therapeutic targets in psychiatric disorders.

## Conflict of Interest Statement

The authors declare that the research was conducted in the absence of any commercial or financial relationships that could be construed as a potential conflict of interest.

## Funding

The presented work is supported by the National Health and Medical Research Council Australia (APP 1043771 to BB). The funders had no role in study design, data collection and analysis, decision to publish, or preparation of the manuscript.

## Appendix

A systematic literature search was performed using the PubMed and Cochrane Library databases covering publications from 1969 to 2015. The following limits were applied: published online, English language, published between 1969 and April 2015. The following search terms were used: [“Chemokines” (Mesh)] OR Chemokine* OR CCL1 OR CCL2 OR CCL3 OR CCL4 OR CCL5 OR CCL6 OR CCL7 OR CCL8 OR CCL9 OR CCL10 OR CCL11 OR CCL12 OR CCL13 OR CCL14 OR CCL15 OR CCL16 OR CCL18 OR CCL19 OR CCL20 OR CCL21 OR CCL22 OR CCL23 OR CCL24 OR CCL25 OR CCL26 OR CCL27 OR CCL28 OR CCR1 OR CCR2 OR CCR3 OR CCR4 OR CCR5 OR CCR62 OR CCR7 OR CCR8 OR CCR9 OR CCR10 OR I-309 OR TCA-3 OR MCP-1 OR MIP-1a OR RANTES OR C10 OR MRP-2 OR C10 OR MRP-2 OR MARC OR MCP-3 OR MCP-2 OR MRP-2 OR CCF18 OR Eotaxin OR MCP-5 OR MCP-4 OR NCC-1 OR Leukotactin-1 OR MIP-5 OR HCC-2 OR NCC-3 OR LEC OR NCC-4 OR LMC OR TARC OR dendrokine OR ABCD-2 OR PARC OR DC-CK1 OR AMAC-1 OR MIP-4 OR ELC OR Exodus-3 OR LARC OR Exodus-1 OR SLC OR 6Ckine OR Exodus-2 OR TCA-4 OR MDC OR MPIF-1 OR MIP-3 OR MPIF-1 OR Eotaxin-2 OR MPIF-2 OR TECK OR Eotaxin-3 OR MIP-4a OR IMAC OR TSC-1 OR CTACK OR ILC OR Eskine OR PESKY OR skinkine OR MEC OR CXCL1 OR CXCL2 OR CXCL3 OR CXCL4 OR CXCL5 OR CXCL6 OR CXCL7 OR CXCL8 OR CXCL9 OR CXCL10 OR CXCL11 OR CXCL12 OR CXCL13 OR CXCL14 OR CXCL15 OR CXCL16 OR CXCL17 OR CXCR1 OR CXCR2 OR CXCR3 OR CXCR4 OR CXCR5 OR CXCR6 OR CXCR7 OR Gro-a OR GRO1 OR NAP-3 OR KC OR Gro-b OR GRO2 OR MIP-2a OR GRO3 OR MIP-2 OR PF-4 OR ENA-78 OR GCP-2 OR NAP-2 OR CTAPIII OR beta-Ta OR PEP OR IL-8 OR NAP-1 OR MDNCF OR GCP-1 OR MIG OR CRG-10 OR IP-10 OR CRG-2 OR I-TAC OR beta-R1 OR IP-9 OR SDF-1 OR PBSF OR BCA-1 OR BLC OR BRAK OR bolekine OR Lungkine OR WECHE OR SRPSOX OR DMC OR VCC-1 OR XCL1 OR XCL2 OR XCR1 OR Lymphotactin a OR SCM-1a OR ATAC OR Lymphotactin b OR SCM-1beta OR CX3CL1 OR CX3CR1 OR Fractalkine OR Neurotactin OR ABCD-3) AND ((Psychiatry*OR psychiatric) OR (Depression OR depress*OR (“Depression”[Mesh]) OR dysthymia OR dysthymi*OR melanchol*OR melancholia OR antidepress*OR (“Antidepressive Agents”[Mesh]) OR ECT OR (“Electroconvulsive Therapy”[Mesh]) OR ((learned AND helplessness) OR (“Helplessness, Learned”[Mesh])) OR “chronic mild stress” OR “chronic unpredictable mild stress” OR “unpredictable chronic mild stress” OR “sucrose preference test” OR “tail suspension test” OR “forced swim test” OR “porsolt test” OR “sickness behavior” OR “olfactory bulbectomy” OR “intracranial self-stimulation”) OR (“Bipolar Disorder” [Mesh] OR bipolar OR mania OR manic) OR (“Schizophrenia”[Mesh] OR schizophreni*OR psychosis OR psychotic OR “prepulse inhibition”)). At this stage, 1847 abstracts were reviewed and studies were excluded if they did not include original data on the measurement of a chemokine and a psychiatric disorder or animal model of a psychiatric disorder. This resulted in 1664 abstracts being excluded. Three articles were also obtained from a review of reference lists. A total of 183 full text manuscripts were retrieved. At this stage, studies were further excluded as per Figure 1. In all, 157 articles remained for this review (see Figure 1).
